# Monomeric C-Reactive Protein Binds and Neutralizes Receptor Activator of NF-κB Ligand-Induced Osteoclast Differentiation

**DOI:** 10.3389/fimmu.2018.00234

**Published:** 2018-02-19

**Authors:** Zhe-Kun Jia, Hai-Yun Li, Yu-Lin Liang, Lawrence Albert Potempa, Shang-Rong Ji, Yi Wu

**Affiliations:** ^1^MOE Key Laboratory of Cell Activities and Stress Adaptations, School of Life Sciences, Lanzhou University, Lanzhou, China; ^2^MOE Key Laboratory of Environment and Genes Related to Diseases, School of Basic Medical Sciences, Xi’an Jiaotong University, Xi’an, China; ^3^Roosevelt University College of Pharmacy, Schaumburg, IL, United States; ^4^The Affiliated Children’s Hospital of Xi’an Jiaotong University, Xi’an, China

**Keywords:** inflammation, rheumatoid arthritis, osteoclast, receptor activator of NF-κB ligand, C-reactive protein

## Abstract

C-reactive protein (CRP) is an established marker of rheumatoid arthritis (RA) but with ill-defined actions in the pathogenesis. Here, we show that CRP regulates the differentiation of osteoclasts, a central mediator of joint inflammation and bone erosion in RA, in a conformation- and receptor activator of NF-κB ligand (RANKL)-dependent manner. CRP in the native conformation is ineffective, whereas the monomeric conformation (mCRP) actively modulates osteoclast differentiation through NF-κB and phospholipase C signaling. Moreover, mCRP can bind RANKL, the major driver of osteoclast differentiation, and abrogate its activities. The binding and inhibition of RANKL are mediated by the cholesterol binding sequence (CBS) of mCRP. Corroborating the *in vitro* results, CRP knockout exacerbates LPS-induced bone resorption in mice. These results suggest that mCRP may be protective in joint inflammation by inhibiting pathological osteoclast differentiation and that the CBS peptide could be exploited as a potential RANKL inhibitor.

## Introduction

Rheumatoid arthritis (RA) is an autoimmune disease characterized by chronic joint inflammation and bone erosion (1). Autoimmunity in RA is likely triggered first at mucosal sites in response to environmental insults but needs to specifically target joints to initiate the disease. Osteoclasts play a major part in homing systemic autoimmunity to joints (2). These cells locate in the bone compartments, and are differentiated from myeloid precursors driven primarily by receptor activator of NF-κB ligand (RANKL) (3). They respond to autoantibodies frequently found in RA by secreting cytokines that evoke and amplify joint inflammation. Moreover, osteoclasts mediate bone resorption directly causing joint damage (4). Therefore, modulating the differentiation and actions of osteoclasts represents a promising strategy for treatment of RA (2, 5, 6).

C-reactive protein (CRP) is an established biomarker of RA (7, 8) with its blood levels closely associated with disease severity and progression (9). However, it remains elusive how CRP acts in the underlying pathological process. CRP appears to be protective in mice with collagen-induced arthritis (10, 11), but fails to influence autoantibody responses (11). This suggests that the protection by CRP might be exerted at joint level. Therefore, recent studies have examined the effects of CRP on the differentiation of osteoclasts, but yielding opposite conclusions (12, 13). Here, we address this controversy by demonstrating the conformation- and RANKL-dependent actions of CRP. Only with the monomeric conformation (mCRP) that is converted specifically at inflammatory loci (14–18), can this protein regulate osteoclast differentiation and neutralize the activities of RANKL. Therefore, exploiting mCRP-RANKL interactions might provide a novel osteoclast-targeting strategy in RA.

## Materials and Methods

### Reagents

Native CRP (nCRP; purity > 97%) purified from human ascites was purchased from the BindingSite (Birmingham, UK; catalog number: BP300.X; lot number: 361639 and 404353) and repurified with p-aminophenyl phosphoryl choline agarose (Thermo Fisher Scientific, Rockford, IL, USA; catalog number: 20307). Wide-type and mutant mCRP were prepared as described (19, 20). Proteins were dialyzed to remove NaN_3_, and passed through Detoxi-Gel Columns (Thermo Fisher Scientific, Rockford, IL, USA; catalog number: 20344) to remove endotoxin when necessary. CRP peptides (purity > 98%) were synthesized by Science Peptide Biological Technology (Shanghai, China). Lyophilized peptides were reconstituted aseptically with DMSO at 40 mg/ml and stored at −20°C in aliquots or kept at 4°C for a maximum of 1 week. Mouse antihuman mCRP mAbs were generated as described (21). 2.5 µg/ml polymyxin B (PMB, Inalco Pharmaceuticals, San Luis Obispo, CA, USA; catalog number: 1758-9325; lot number: R1/51/121) was included in all cell response experiments to neutralize residual endotoxin.

### Osteoclast Differentiation

RAW 264.7 murine macrophage cells (13) and bone marrow-derived macrophages (BMDMs) (22) were prepared and cultured as described. Briefly, bone marrow cells were isolated from femurs and tibias of 6-week-old male C57BL/6 mice. Following removal of red blood cells, the remaining cells were cultured in α-MEM (Hyclone, Logan, UT, USA; catalog number: SH30265.01; lot number: AC10207087, AB10164750) containing 10% FBS and 5 ng/ml M-CSF (R&D Systems, Minneapolis, MN, USA; catalog number: 416-ML; lot number: ME2915062 and ME3919011) for 12 h. Nonadherent cells were cultured for another 3 days with 10 ng/ml M-CSF. After vigorous washing, adherent cells were harvested and seeded in 24-well plates for 1–2 days and used as BMDMs. 10 ng/ml M-CSF was copresent in all treatments of BMDMs.

Raw cells or BMDMs were treated with nCRP, mCRP, RANKL (R&D Systems, Minneapolis, MN, USA; catalog number: 462-TEC; lot number: CWA1815111), or their combinations to induce osteoclast differentiation. Culture media were changed every two days. In some experiments, BMDM were pretreated with signaling inhibitors and then stimulated with mCRP or RANKL for 24 h. The used inhibitors were: Bay11-7082 (10 µM, 1 h pretreatment; NF-κB inh), SB20358 (20 µM, 15 min pretreatment; p38 MAPK inh), SP600125 (10 µM, 1 h pretreatment; JNK inh), U0126 (10 µM, 1 h pretreatment; ERK inh), U73122 [10 µM, 0.5 h pretreatment; phospholipase C (PLC) inh], LY294002 (50 µM, 1 h pretreatment; PI3K inh), MK-2206 2HCl (5 µM, 1 h pretreatment; Akt inh), Piceatannol (10 µM, 1 h pretreatment; Syk inh), and GW5074 (20 µM, 0.5 h pretreatment; Raf inh).

Total RNA was extracted with RNAiso Plus reagent (Takara, Shiga, Japan; catalog number: 9109; lot number: AKA3402, AKA5802). cDNA was synthesized from 2 µg total RNA using PrimeScript RT Master Mix system (Takara; catalog number: RR036A; lot number: AK4102, AK4403). The expression of osteoclast marker genes was determined with quantitative PCR using RealStar Green Power Mixture (Genestar, Beijing, China; catalog number: A311; lot number: 7AB01) in a StepOne Plus real-time PCR system (Thermo Fisher Scientific). The gene expression levels were normalized to that of GAPDH. The primer sequences used were: TRAP (forward: 5′-GCAACATCCCCTGGTATGTG-3′; reverse: 5′-GCAAACGGTAGTAAGGGCTG-3′); Cathepsin K (forward: 5′-GCATTACCAACATGGCCAGC-3′; reverse: 5′-CTCCCTTCCAAAGCCACCAA-3′); RANKL (forward: 5′-CAGCATCGCTCTGTTCCTGTA-3′; reverse: 5′-CTGCGTTTTCATGGAGTCTCA-3′); NFKB2 (forward: 5′-GGCCGGAAGACCTATCCTACT-3′; reverse: 5′-CTACAGACACAGCGCACACT-3′); SOCS1 (forward: 5′-CTGCGGCTTCTATTGGGGAC-3′; reverse: 5′-AAAAGGCAGTCGAAGGTCTCG-3′); SOCS3 (forward: 5′-ATGGTCACCCACAGCAAGTTT-3′; reverse: 5′-TCCAGTAGAATCCGCTCTCCT-3′); PPARG (forward: 5′-GGAAGACCACTCGCATTCCTT-3′; reverse: 5′-GTAATCAGCAACCATTGGGTCA-3′); GAPDH (forward: 5′-GGGCTACACTGAGGACCAGGTT-3′; reverse: 5′-TGCTGTAGCCGTATTCATTGTCA-3′).

Gene expression profiles were determined with SurePrint G3 Mouse Gene Expression 8*60K Microarray (Agilent Technologies, Santa Clara, CA, USA) by CapitalBio Technology (Beijing, China). Expression ratios were calculated, first normalized to the 75th percentile per chip, and finally normalized to medians per gene.

Following differentiation for 6 days, cells were fixed with formaldehyde and the number of osteoclasts was determined with a TRAP staining kit (Sigma-Aldrich, St. Louis, MO, USA; catalog number: 387A-1KT; lot number: SLBP7795V) according to the manufacture’s instruction. TRAP-positive multinucleated cells (>3 nuclei/cell) were counted by a light microcopy as osteoclasts.

### Bone Resorption

RAW cells or BMDMs were seeded on fresh bovine femur slices of 20-µm thick in 24-well plates. Osteoclast differentiation was induced by treatment of nCRP, mCRP, RANKL or their combinations with culture media changed every 2 days. Six days later, the slices were washed with 1 M ammonia for 3 min to break the osteoclasts, then fixed with 50% glutaric acid for 3 min, and finally stained with 1% toluidine blue for 5–10 min. Bone resorption was measured by pit area.

### RANKL Binding

The interaction of mCRP and RANKL was determined with ELISA. Briefly, microtiter wells were coated with 1 µg/ml RANKL overnight at 4°C. All the following steps were performed at 37°C, and after each incubation step wells were washed 3 times with TBS (10 mM Tris, 140 mM NaCl, pH 7.4) containing 0.02% NP-40. Wells were washed and blocked with 1% BSA in TBS. nCRP, mCRP, or mCRP mutant was added to immobilized RANKL for 1 h followed by washing. Binding were then detected with a sheep antihuman CRP polyclonal antibody (BindingSite; catalog number: PC044; lot number: 352325) and a donkey antisheep IgG (H + L) secondary antibody (Abbkine, Wuhan, China; catalog number: A21060; lot number: ATQMA0601).

### Inflammatory Osteolysis

CRP knockout (KO) mice of C57BL/6 background were generated by insertion of a floxed STOP cassette at the translation start site of CRP gene *via* homologous recombination using CRISPR/Cas9 technique (Shanghai Biomodel Organism Science & Technology Development, Shanghai, China). CRP KO mice are fertile and grossly healthy. Inflammatory osteolysis was induced in wild-type or CRP KO mice (25 ± 2 g) of 7–8 weeks age as described (23). Briefly, 5 mg/kg LPS (Sigma-Aldrich; catalog number: L2880, lot number: 25M4040V), 2.5 mg/kg mCRP or vehicle was injected into the subcutaneous tissues overlying calvaria. The injections were performed every other day for 7 days. The calvaria were harvested and fixed in 4% paraformaldehyde for 2 days, followed by decalcification with 10% neutral buffered EDTA and embedding in paraffin. Samples were sectioned and TRAP and hematoxylin and eosin (HE) staining were performed to evaluate osteoclastogenesis and bone damage. The experiments conformed to the Guide for the Care and Use of Laboratory Animals published by NIH and were conducted according to the protocols approved by the Ethics Committee of Animal Experiments of Xi’an Jiaotong University.

### Fluorescence Imaging

Raw264.7 and BMDM cells cultured on coverslips were rinsed twice with sterile PBS and incubated with FITC-labeled nCRP or mCRP for 30 min at 4°C. After gently rising, cell membrane was marked with FM 4-64 (Invitrogen, Carlsbad, CA, USA; catalog number: F34653; lot number: 1814727) at 4°C for 1 min. Nuclei were counterstained with DAPI (SouthernBiotech, Birmingham, AL, USA; catalog number: 0100-20; lot number: F0617-S327). Samples were examined by a LSM 710 confocal microscopy (Zeiss, Jena, Germany).

### Statistical Analysis

Data were presented as mean ± SEM. Statistical analysis was performed by two-tailed Student’s *t*-test, one-way ANOVA with Tukey *post hoc* or Kolmogorov–Smironv tests as appropriate. Values of *p* < 0.05 were considered significant.

## Results

### mCRP Induces Osteoclast Differentiation

**Figure 1:**
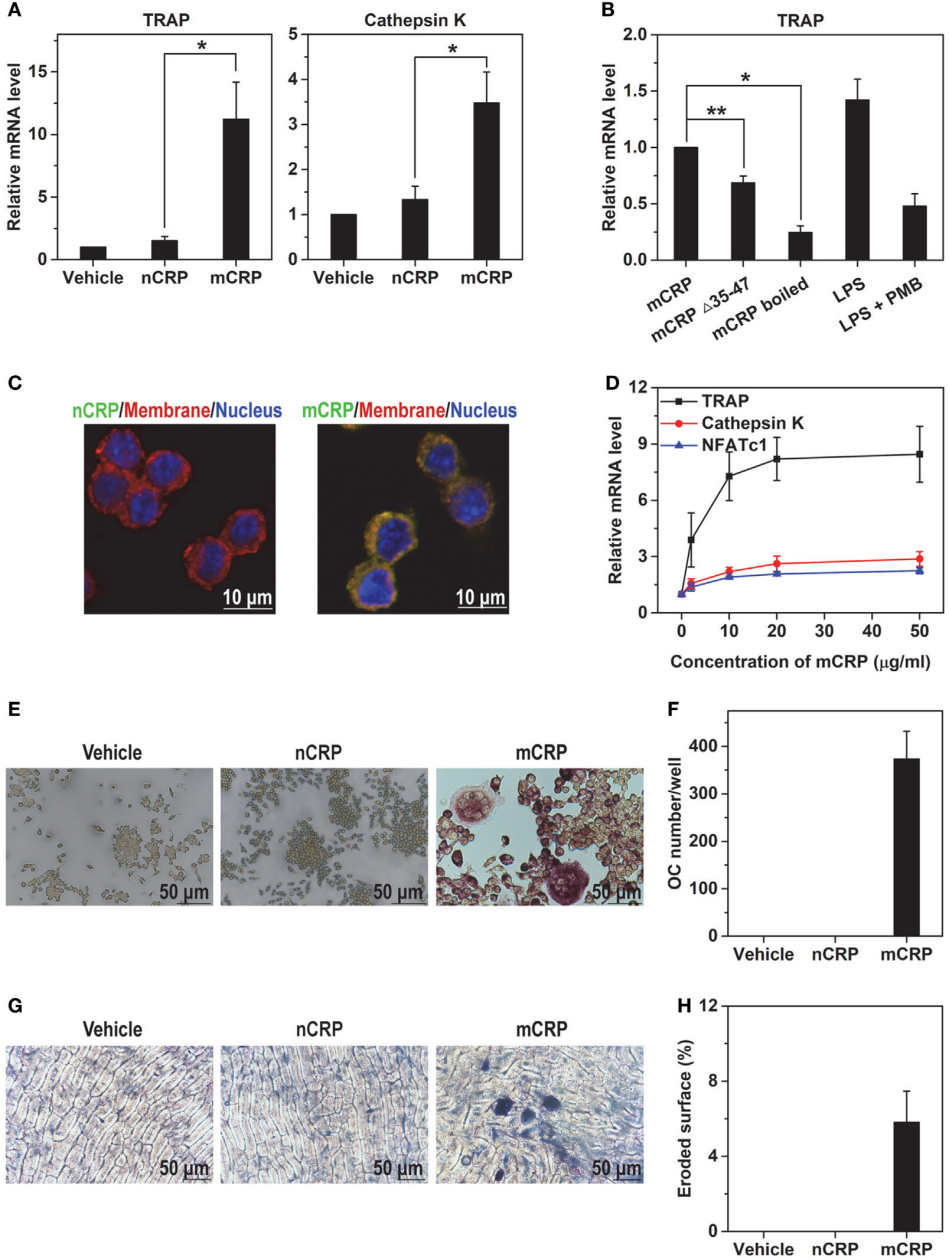
mCRP but not native C-reactive protein (nCRP) induces osteoclast differentiation of Raw 264.7 cell line. Raw cells were treated with the indicated reagents for 2 days and the expression of osteoclast marker genes was determined with q-PCR. **(A)** 100 µg/ml mCRP upregulated the expression of TRAP and Cathepsin K, while nCRP at the same concentration was ineffective. **(B)** mCRP mutant lacking cholesterol-binding sequence motif (Δ35–47) or boiled wild-type mCRP showed impaired capacity to upregulate the expression of TRAP. The effects of LPS at 100 ng/ml, which is 50-fold higher than the residual level of endotoxin in our mCRP preparation, could be abrogated by the copresence of 2.5 µg/ml polymyxin B (PMB) that was included in all differentiation experiments. **(C)** FITC-labeled nCRP and mCRP were incubated with Raw cells at 4°C and visualized by confocal microscopy. Cell membranes and nuclei were counterstained with FM-4-64 and DAPI, respectively. mCRP showed intense binding to Raw cells, but nCRP did not. **(D)** Dose-dependent induction of TRAP expression by mCRP. Following treatment with 100 µg/ml nCRP or mCRP for 6 days, Raw cells were stained for TRAP **(E)** and osteoclast number was counted as TRAP-positive multinucleated cells **(F)**. Raw cells were plated on bone slices and treated with nCRP or mCRP for 6 days. The slices were then stained by toluidine blue **(G)** to measure eroded surface **(H)**. mCRP but not nCRP induced the differentiation of Raw cells to mature osteoclasts with bone resorption activities.

Circulating CRP is composed of five identical subunits, but dissociates into the monomeric conformation, i.e., mCRP, upon entering local lesions (14–18). Indeed, mCRP has been identified as the major conformation present in synovium tissues of RA patients (24). CRP in different conformations exhibit distinct or even contrasting activities (14–18), which may account for the controversies on its role in osteoclast differentiation (12, 13). We thus first examined this issue using Raw 264.7 macrophage cell line. Treating Raw cells with nCRP for 2 days did not alter the expression of osteoclast maker genes TRAP (also called ACP5) and Cathepsin K (Figure 1A). By contrast, these genes were markedly upregulated by mCRP treatment.

The effects of mCRP were not due to endotoxin contaminant because experiments were performed in the presence of PMB. Moreover, boiling or deleting the key recognition motif, i.e., cholesterol-binding sequence (CBS; a.a. 35–47) (20) that interacts with the lipid raft receptor (25, 26), impaired the actions of mCRP (Figure 1B). mCRP also showed much stronger binding to Raw cells than nCRP (Figure 1C), and evoked substantial responses at a concentration of 2 µg/ml (Figure 1D). The capability of mCRP to drive osteoclast differentiation was further confirmed by functional assays of TRAP staining (Figures 1E,F) and bone resorption (Figures 1G,H), in which nCRP was ineffective.

**Figure 2:**
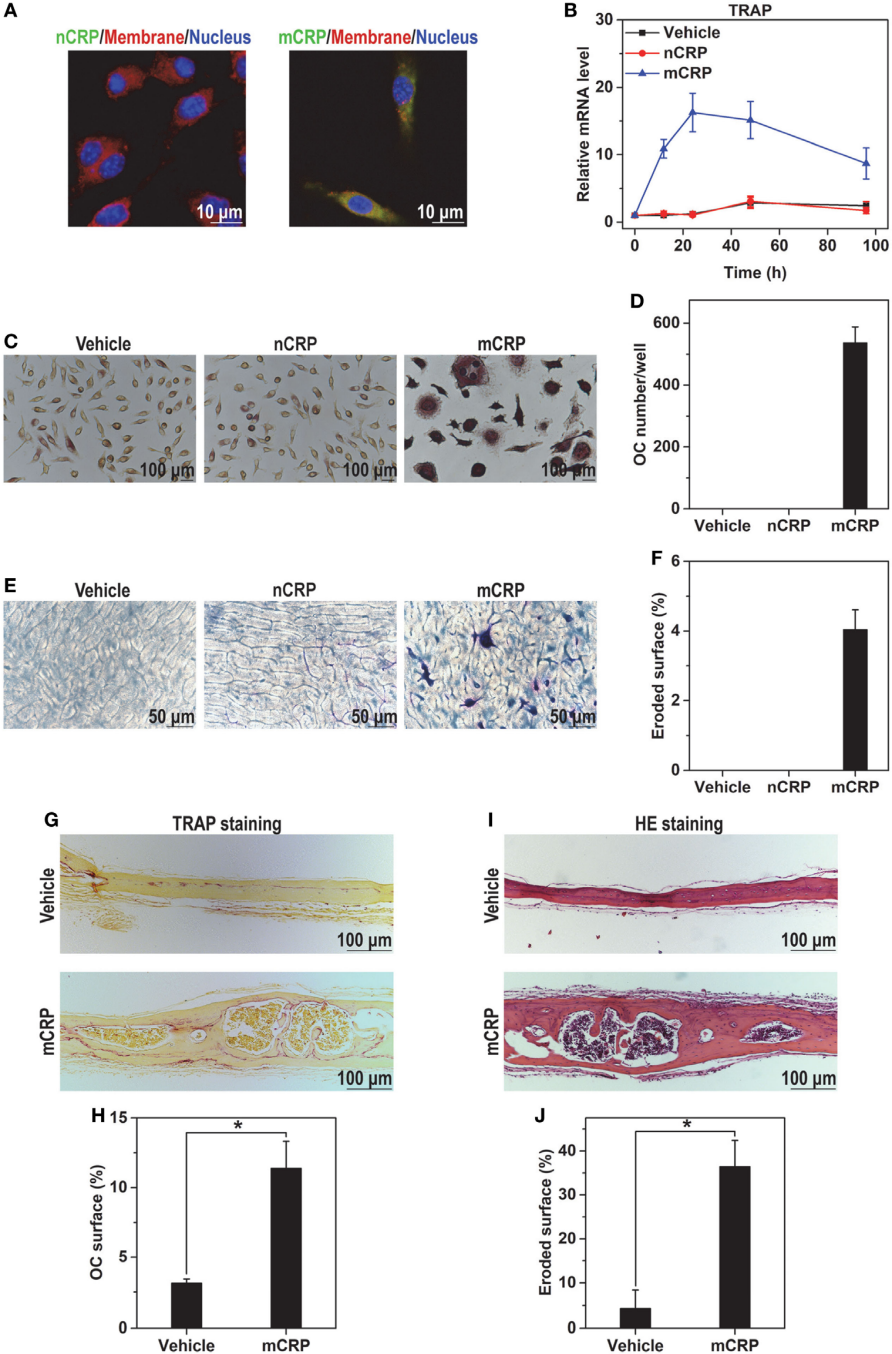
mCRP but not native C-reactive protein (nCRP) induces osteoclast differentiation of bone marrow-derived macrophages (BMDMs) and in mice. **(A)** FITC-labeled nCRP or mCRP was incubated with BMDMs at 4°C and visualized by confocal microscopy. Cell membranes and nuclei were counterstained with FM-4-64 and DAPI, respectively. mCRP bound BMDMs strongly, while nCRP did not. **(B)** BMDMs were treated with 100 µg/ml nCRP or mCRP for the indicated times. TRAP expression was induced by mCRP but not nCRP. BMDMs were treated with 100 µg/ml nCRP or mCRP for 6 days, and then stained for TRAP **(C)** to count the number of TRAP-positive multinucleated cells as osteoclasts **(D)**. BMDMs were plated on bone slices and treated with nCRP or mCRP for 6 days. The slices were then stained by toluidine blue **(E)** to measure eroded surface **(F)**. mCRP but not nCRP induced the differentiation of BMDMs to mature osteoclasts with bone resorption activities. mCRP (2.5 mg/kg) or saline buffer (Vehicle) was s.c. injected on calvaria of healthy wild-type mice every 2 days for 1 week. TRAP **(G,H)** and hematoxylin and eosin staining **(I,J)** were conducted to evaluate osteoclastogenesis and bone damage, respectively. Osteolysis was actively induced by mCRP injection.

We next validated the conformation-dependent actions of CRP using mouse BMDMs. nCRP showed only weak binding to BMDMs and was unable to drive osteoclast differentiation; while mCRP bound BMDMs intensely (Figure 2A) and induced strong expression of TRAP (Figure 2B), leading to the formation of multinucleated osteoclasts (Figures 2C,D) with bone resorption activities (Figures 2E,F). Consistent with the *in vitro* results, subcutaneous injection of mCRP on calvaria of healthy mice resulted in increased number of osteoclasts (Figures 2G,H) and obvious trabecular damage (Figures 2I,J). We thus conclude that the induction of osteoclast differentiation by CRP depends on the monomeric conformation.

### mCRP Does Not Act *via* Induction of RANKL

**Figure 3:**
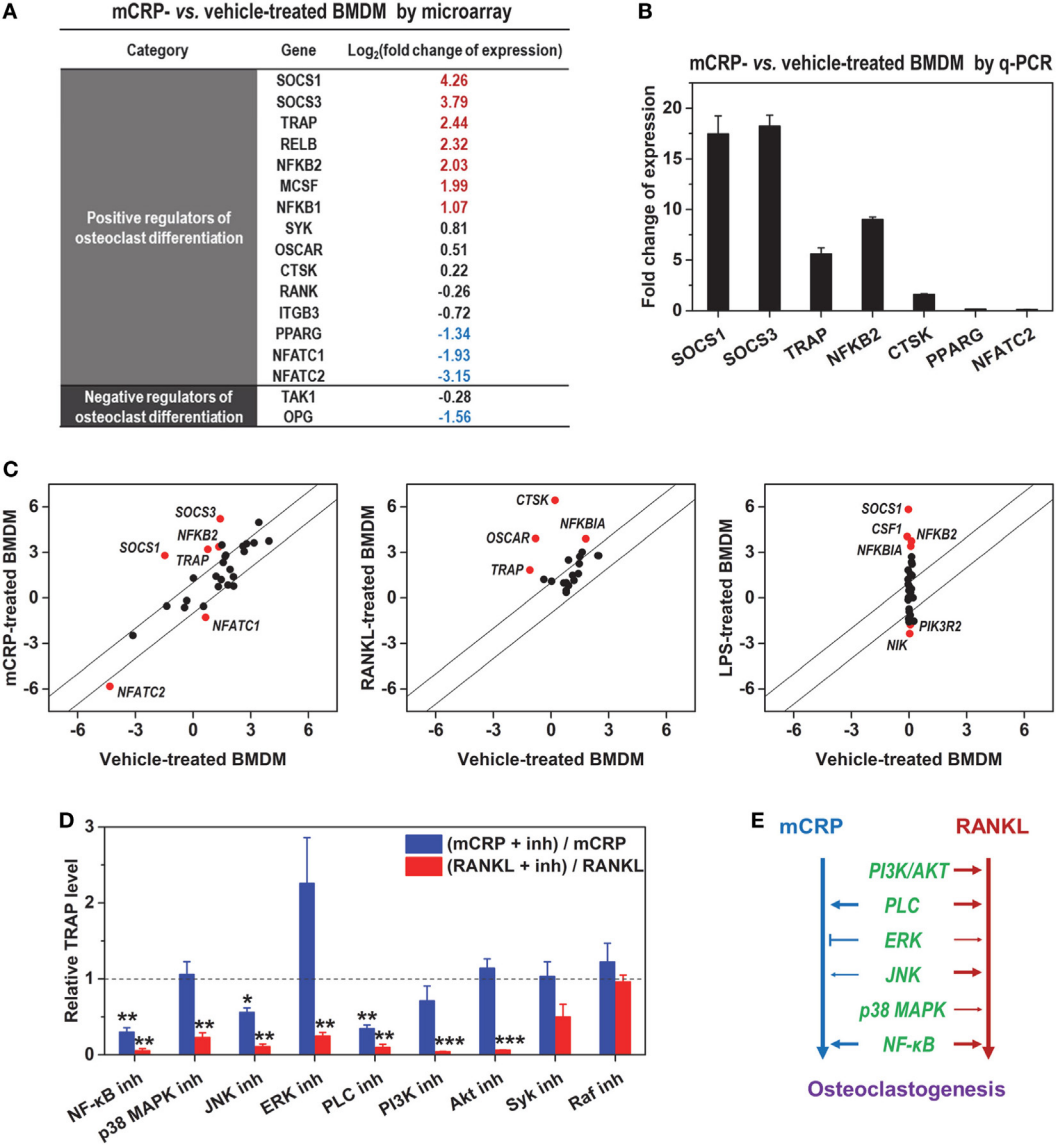
mCRP acts *via* receptor activator of NF-κB ligand (RANKL)-independent pathway. **(A)** The expression profiles of mCRP- and vehicle-treated bone marrow-derived macrophages (BMDMs) were determined with DNA microarray. The expression changes of confidently determined genes relevant to osteoclast differentiation were listed. mCRP did not induce the expression of RANKL or its activating receptor RANK. **(B)** q-PCR validation of gene expression induced by mCRP. **(C)** Comparison of the effects of mCRP, RANKL (GSE57468), and LPS (GSE21895) on the expression of osteoclastogenesis-relevant genes. Remarkable differences in their effects were evident. **(D)** BMDMs were treated with mCRP or RANKL in the presence or absence of the indicated signaling inhibitors: Bay11-7082 (10 µM, NF-κB inh), SB20358 (20 µM, p38 MAPK inh), SP600125 (10 µM, JNK inh), U0126 (10 µM, ERK inh), U73122 (10 µM, Phospholipase C (PLC) inh), LY294002 (50 µM, PI3K inh), MK-2206 2HCl (5 µM, Akt inh), Piceatannol (10 µM, Syk inh), and GW5074 (20 µM, Raf inh). The mRNA levels of TRAP were determined by q-PCR and normalized to that of controls treated only with inhibitor. Results are shown as (TRAP level of cells treated with mCRP or RANKL plus the indicated inhibitor)/(TRAP level of cells treated with mCRP or RANKL). **(E)** The signaling pathways evoked by the two inducers differed significantly.

RANKL is considered as the major inducer of osteoclast differentiation (3) and has been reported to be upregulated by CRP (12). To clarify whether the effects of mCRP are mediated *via* downstream RANKL, we performed expression profiling on mCRP-treated BMDMs by DNA microarray. Osteoclast differentiation emerged as one of the top-ranked pathways activated by mCRP. Of the 35 relevant genes, 11 were differentially expressed in response to mCRP following a 4-h treatment (Figures 3A,B). Importantly, mCRP did not induce the expression of RANKL, its activating receptor RANK, or the inhibitory receptor LGR4 (22), but downregulated the expression of the decoy receptor OPG, suggesting little involvement of the canonical RANKL pathway in mediating the downstream effects of mCRP.

Additional analysis revealed remarkable differences between the gene expression profiles induced by mCRP and RANKL (Figure 3C). Moreover, screening with signaling inhibitors demonstrated that they activated distinct pathways (Figure 3D). mCRP acted primarily through NF-κB and phospholipase C, while a more extensive network was involved in RANKL signaling (Figure 3E). Of particular interest, ERK inhibition with U0126 markedly enhanced the effects of mCRP but suppressed that of RANKL. These results together demonstrate that the induction of osteoclast differentiation by mCRP does not depend on RANKL.

### mCRP Inhibits RANKL-Induced Osteoclast Differentiation

**Figure 4:**
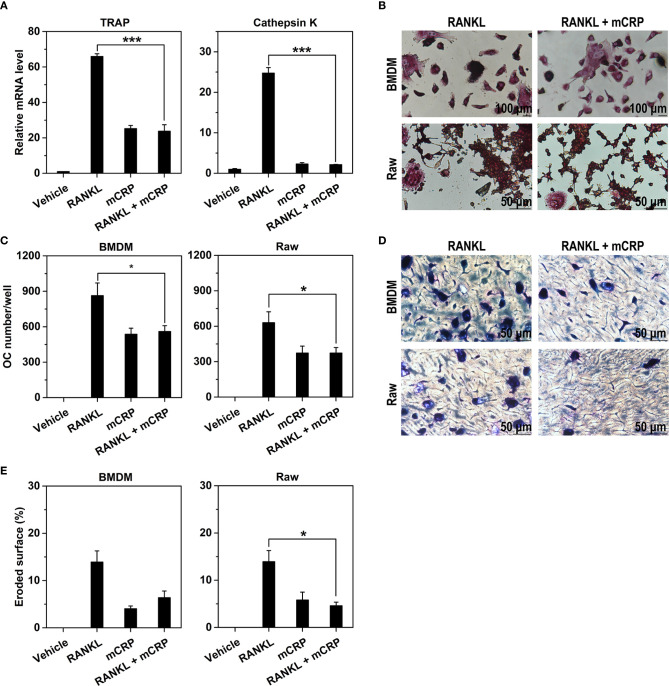
mCRP neutralizes the effects of receptor activator of NF-κB ligand (RANKL). Bone marrow-derived macrophages (BMDMs) were treated with 10 ng/mL of M-CSF and 50 ng/ml RANKL in the presence or absence of 100 µg/mL of mCRP. After treatment for 2 days, the expression of TRAP and Cathepsin K were determined by q-PCR **(A)**. After treatment for 6 days, cells were stained for TRAP **(B)** and counted for the number of osteoclasts **(C)**. Their bone resorption activities were evaluated by toluidine blue staining **(D)** and the quantification of eroded surface **(E)**. The potent effects of RANKL on BMDMs were absent when treated together with mCRP. Comparable results were also obtained with Raw cells **(B–E)**. *p < 0.05; ***p < 0.001.

As mCRP appeared to act differently from RANKL, we asked whether these two inducers have any synergy in osteoclast differentiation. When applied alone, RANKL was 3–10-fold more potent than mCRP in inducing the expression of osteoclast maker genes in BMDMs (Figure 4A). When applied together with mCRP, however, the effects of RANKL was completely absent with the net responses comparable to that of mCRP acting alone. Similar findings were also obtained in TRAP staining (Figures 4B,C) and bone resorption assays (Figures 4D,E). These results suggest that mCRP might instead inhibit the activities of RANKL on osteoclast differentiation.

**Figure 5:**
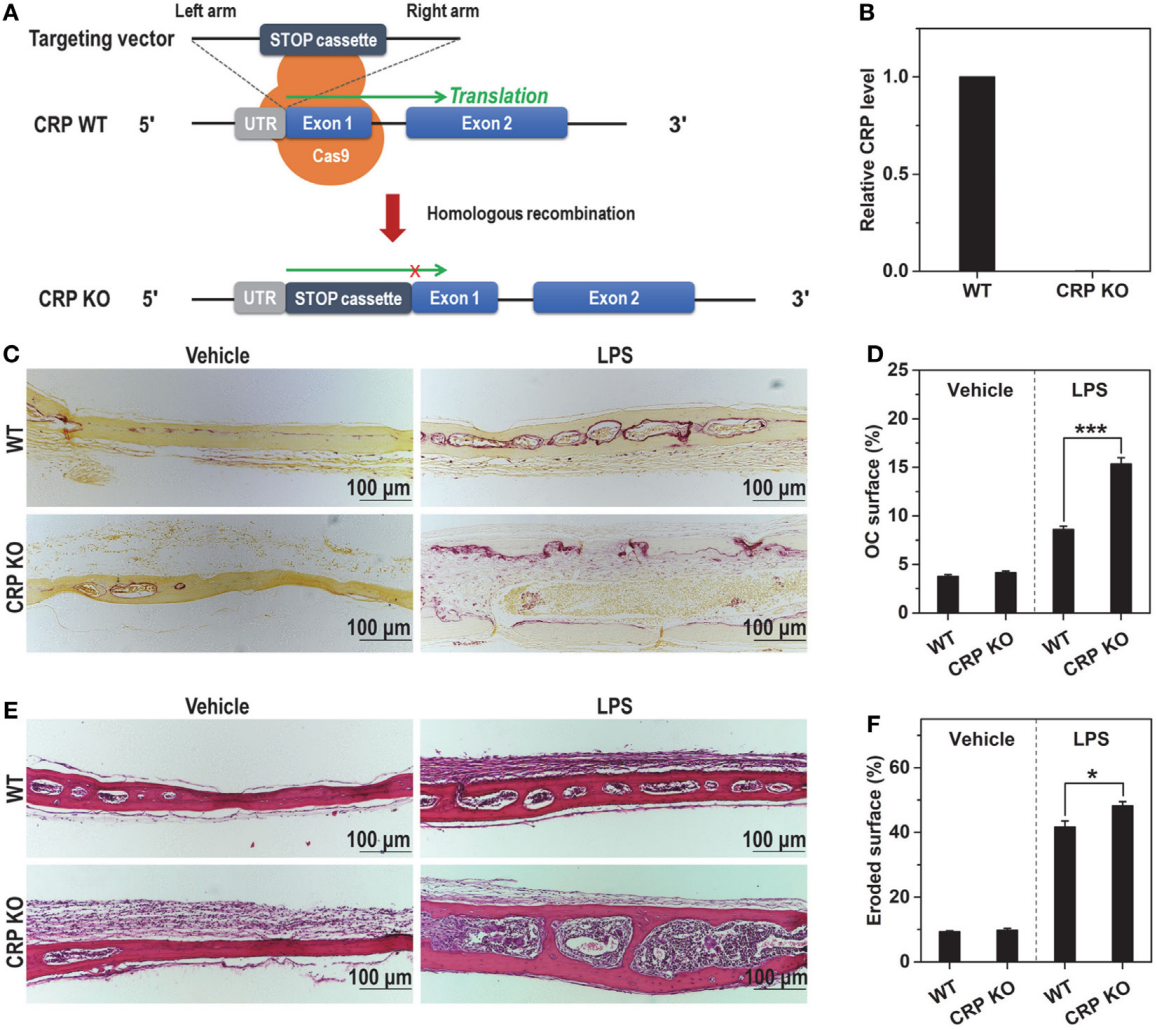
C-reactive protein (CRP) knockout (KO) aggravates proinflammatory bone damage in mice**. (A)** The design of CRP KO mice. **(B)** The mRNA level of CRP in liver tissues of wild-type (WT) and CRP KO mice. No CRP expression could be detected in CRP KO mice. LPS (5 mg/kg) or saline buffer (Vehicle) was s.c. injected on calvaria of healthy wild-type and CRP KO mice every 2 days for 1 week. TRAP **(C,D)** and hematoxylin and eosin staining **(E,F)** were conducted to evaluate osteoclastogenesis and bone damage, respectively. These pathological indices were aggravated in CRP KO versus wide type mice.

mCRP is specifically converted in inflamed tissues, but does not exist in normal tissues (14–18). Therefore, it is plausible that the actions of mCRP are confined to pathological osteoclast differentiation where overproduced RANKL also plays a predominant role (4–6). The overall effects of mCRP in such a scenario, however, are likely suppressive due to inhibition of RANKL. In line with this speculation, the bone resorption phenotype of CRP KO mice (Figures 5A,B) is indistinguishable from that of wild-type mice in normal calvaria, but manifested increased number of osteoclasts (Figures 5C,D) and enhanced damage of trabeculae (Figures 5E,F) following subcutaneous injection of LPS.

### CBS Mediates the Binding and Inhibition of RANKL

**Figure 6:**
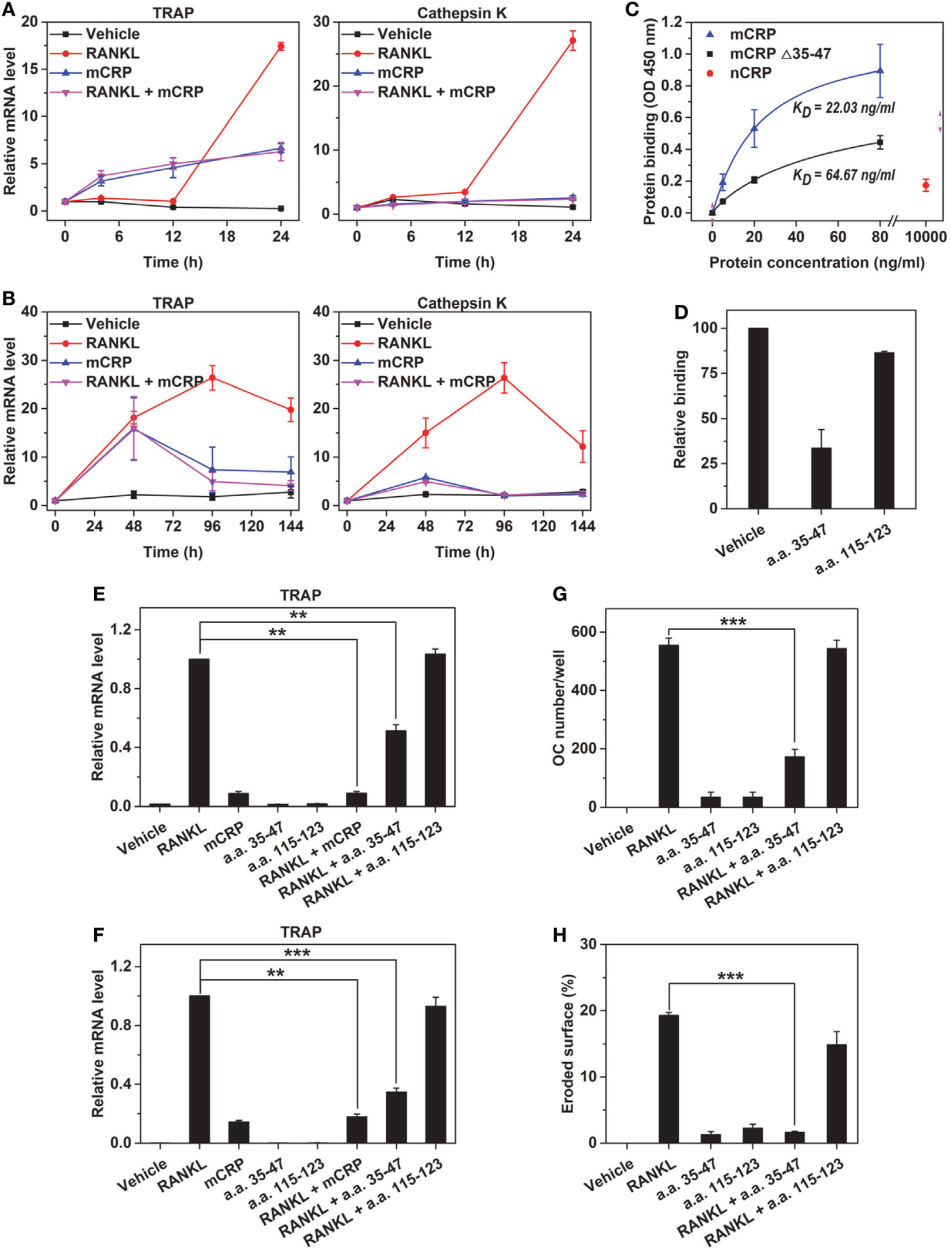
mCRP binds receptor activator of NF-κB ligand (RANKL) *via* cholesterol-binding sequence. Bone marrow-derived macrophages (BMDMs) **(A)** or peripheral blood mononuclear cells **(B)** were treated with RANKL, mCRP, or their combination for the indicated times. In both cell types, the effects of RANKL were abrogated by cotreatment of mCRP at all time points tested. **(C)** mCRP, mCRP Δ35–47, or native C-reactive protein (nCRP) at the indicated concentrations were added to RANKL immobilized on microtiter wells. mCRP avidly bound immobilized RANKL, but this binding was impaired by deleting a.a. 35–47. Weak binding of nCRP was only detected at very high concentration. a.a. 35–47 peptide (20 µg/ml) nearly abrogated the binding of mCRP to RANKL **(D)**, halved RANKL-induced TRAP expression in Raw **(E)** and BMDM **(F)**, and greatly suppressed RANKL-induced differentiation of osteoclasts **(G)** and their bone resorption activities **(H)**. By contrast, another CRP peptide, i.e., a.a. 115–123 (20 µg/ml), showed no effect in these assays.

The inhibition of RANKL by mCRP could be due to interference between signaling pathways evoked by them. However, the complete and persistent inhibition observed in mouse BMDMs (Figure 6A) and peripheral blood mononuclear cells (PBMCs) (Figure 6B) made this possibility unlikely. Rather, inhibition due to their direct physical interactions appeared more likely. Indeed, mCRP bound RANKL with high affinity (Figure 6C). Deleting CBS (a.a. 35–47), the major recognition motif of mCRP (20), or competing with the synthetic CBS peptide markedly impaired the binding (Figure 6D). Moreover, the CBS peptide efficiently inhibited RANKL-induced osteoclastogenesis (Figures 6E–H). Therefore, the physical interactions between mCRP and RANKL are mediated by CBS.

## Discussion

The effects of CRP on osteoclast differentiation have been controversial. One study reports that CRP inhibits RANKL-induced osteoclast differentiation of Raw 264.7 cells *via* TLR signaling (13). However, residual endotoxin is a notorious contaminant that confuses the interpretation of CRP’s activity (27, 28). Another study reports that CRP promotes osteoclast differentiation of human PBMCs *via* induction of RANKL (12). However, the prolonged incubation time (3–21 days) and the prominent death of primary cells cultured *ex vivo* would favor the conversion of nCRP to mCRP (29–34). Interestingly, the authors claim that the actions of CRP are mediated at least partly by CD16 (12), an established receptor for mCRP (35).

In the present study, with tight control of endotoxin contaminant and protein conformation, we show that CRP in its native conformation, i.e., nCRP, has no effect on osteoclast differentiation of Raw 264.7 cells, mouse BMDMs or PBMCs; while mCRP actively regulates their differentiation. The remarkable difference between gene expression profiles induced by mCRP and LPS further exclude possible confounding of endotoxin. Our findings thus establish that the effects of CRP on osteoclast differentiation are conformation-dependent. This may account for the aforementioned controversy and argues that conformation control is critical for interpreting the actions of CRP.

The actions of mCRP in osteoclastogenesis appear to depend on crosstalk among NF-κB, phospholipase C and ERK signaling pathways. Indeed, NF-κB, phospholipase C, and p38 MAPK have been shown to be responsible for mCRP-induced cytokine induction in endothelial cells (36, 37), while ERK and PI3K/Akt are more important in mediating the effects of mCRP on angiogenesis (38–41) and survival of neutrophils (42). These findings reveal a cell type- and biological process-dependent signaling evoked by mCRP. Such a versatility in activating various signaling pathways is likely duo to the capacity of mCRP to interact with lipid rafts (25, 26), signaling platforms on cell surfaces. Therefore, adaptor(s) in lipid rafts that mediates the context-specific actions of mCRP warrants further investigation.

It has been increasingly recognized that the actions of CRP also depend on localization (14–18). nCRP circulates in the blood as a pentamer, but undergoes irreversible conformation changes, forming mCRP in inflamed tissues due to interaction with damaged membranes (29–34, 43–46), amyloid aggregates (47), neutrophil extracellular traps (48), or acidic pH (49). mCRP exhibits markedly enhanced activities, and is consequently considered to be the major conformation acting in local lesions (14–18), such as synovium tissues of RA patients (24). Therefore, the interaction of mCRP with osteoclast precursors would be expected to occur in inflamed but not normal joints.

In inflamed joints, dysregulated RANKL signaling likely plays a major role in driving pathological osteoclast differentiation (4–6), and its neutralizing mAb, Denosumab, has become an approved therapeutic (50) that prevents bone loss in RA (51). As such, the overall effects of mCRP in pathological osteoclast differentiation may be protective through antagonizing RANKL. This speculation is supported by exacerbated inflammatory bone resorption in CRP KO versus wild-type mice, and is also consistent with the beneficial role of CRP in collagen-induced arthritis (10, 11). In this regard, CBS that mediates the interaction of mCRP with RANKL and exhibits anti-inflammatory activities as a synthetic peptide (20, 52), might emerge as a potential RANKL inhibitor to target pathological osteoclast differentiation.

Though mCRP is a potent proinflammatory molecule, it also possesses anti-inflammatory actions (53, 54). Therefore, the net contribution of mCRP in diseases might be context-dependent. Indeed, a protective role of mCRP in early atherogenesis has been reported (55). Recently, we have demonstrated that mCRP may be protective in lupus nephritis by recruiting CFH, and autoantibodies against CBS abrogating this effect predicts worse prognosis (52). Of note, dysregulated complement activation is critically involved in both atherosclerosis and lupus nephritis, which may explain the beneficial role of mCRP. In RA, however, dysregulated osteoclast differentiation is a central pathogenic mechanism (2) and mCRP may thus exert protective effects by neutralizing RANKL-induced osteoclastogenesis.

## Ethics Statement

The experiments conformed to the Guide for the Care and Use of Laboratory Animals published by NIH and were conducted according to the protocols approved by the Ethics Committee of Animal Experiments of Xi’an Jiaotong University.

## Author Contributions

YW and S-RJ designed the research. Z-KJ, H-YL, and Y-LL performed the research. YW, S-RJ, Z-KJ, H-YL, and LP analyzed the data and wrote the article. All authors reviewed the results and approved the final version of the manuscript.

## Conflict of Interest Statement

The authors declare that the research was conducted in the absence of any commercial or financial relationships that could be construed as a potential conflict of interest. The reviewer JA and handling editor declared their shared affiliation.
