# Corrigendum: Monomeric C-Reactive Protein Binds and Neutralizes Receptor Activator of NF-κB Ligand-Induced Osteoclast Differentiation

**DOI:** 10.3389/fimmu.2020.619847

**Published:** 2020-11-24

**Authors:** Zhe-Kun Jia, Hai-Yun Li, Yu-Lin Liang, Lawrence A. Potempa, Shang-Rong Ji, Yi Wu

**Affiliations:** ^1^ MOE Key Laboratory of Cell Activities and Stress Adaptations, School of Life Sciences, Lanzhou University, Lanzhou, China; ^2^ MOE Key Laboratory of Environment and Genes Related to Diseases, School of Basic Medical Sciences, Xi’an Jiaotong University, Xi’an, China; ^3^ Roosevelt University College of Pharmacy, Schaumburg, IL, United States; ^4^ The Affiliated Children’s Hospital of Xi’an Jiaotong University, Xi’an, China

**Keywords:** inflammation, rheumatoid arthritis, osteoclast, receptor activator of NF-κB ligand, C-reactive protein

In the original article, there was a mistake in **Figure 4B** as published. The lower panel of **Figure 4B** was erroneously assigned. The corrected **Figure 4B** appears below.

**Figure 4:**
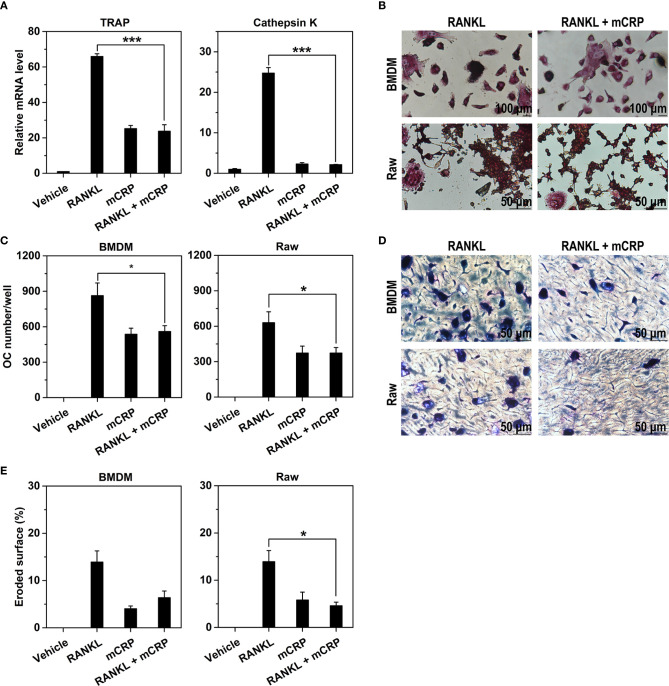
mCRP neutralizes the effects of receptor activator of NF-κB ligand (RANKL). Bone marrow-derived macrophages (BMDMs) were treated with 10 ng/mL of M-CSF and 50 ng/ml RANKL in the presence or absence of 100 μg/mL of mCRP. After treatment for 2 days, the expression of TRAP and Cathepsin K were determined by q-PCR **(A)**. After treatment for 6 days, cells were stained for TRAP **(B)** and counted for the number of osteoclasts **(C)**. Their bone resorption activities were evaluated by toluidine blue staining **(D)** and the quantification of eroded surface **(E)**. The potent effects of RANKL on BMDMs were absent when treated together with mCRP. Comparable results were also obtained with Raw cells **(B–E)**. *p < 0.05; ***p < 0.001.

The authors apologize for this error and state that this does not change the scientific conclusions of the article in any way. The original article has been updated.

